# Signs of Chronic Hypoxia Suggest a Novel Pathophysiological Event in α‐Synucleinopathies

**DOI:** 10.1002/mds.28229

**Published:** 2020-09-03

**Authors:** Antonio Heras‐Garvin, Christoph Danninger, Sabine Eschlböck, Janice L. Holton, Gregor K. Wenning, Nadia Stefanova

**Affiliations:** ^1^ Division of Neurobiology, Department of Neurology Medical University of Innsbruck Innsbruck Austria; ^2^ Division of Neuropathology, UCL Queen Square Institute of Neurology University College London London UK

**Keywords:** multiple system atrophy, Parkinson's disease, α‐synucleinopathies, hypoxia

## Abstract

**Background:**

Multiple system atrophy (MSA) and Parkinson's disease (PD) patients develop respiratory and cardiovascular disturbances including obstructive sleep apnea, orthostatic hypotension, and nocturnal stridor. We hypothesized that, associated with these respiratory and cardiovascular disturbances, hypoxic events may occur in MSA and PD brains that may play a role in disease progression. The objective of this study was to evaluate the presence of hypoxia in nonneurological controls and PD and MSA patients.

**Methods:**

Molecular levels of hypoxia markers were measured in postmortem brain tissue from controls and PD and MSA cases.

**Results:**

MSA brain showed signs of chronic hypoxia characterized by the significant accumulation of the hypoxic marker HIF2α as compared to PD patients and controls. We detected no differences between MSA subtypes. Signs of hypoxia were also observed in PD patients with a clinical presentation similar to the MSA cases.

**Conclusions:**

The results obtained from this study suggest a new alternative pathway associated with α‐synucleinopathies that may contribute to the pathogenesis of these disorders. © 2020 The Authors. *Movement Disorders* published by Wiley Periodicals LLC on behalf of International Parkinson and Movement Disorder Society

Multiple system atrophy (MSA) is a fatal progressive atypical parkinsonian disorder leading to severe motor disability and death a few years after symptom onset.^1^ Based on its clinical presentation, MSA is subdivided in 2 main variants: parkinsonian (MSA‐P) and cerebellar (MSA‐C).^1^ Postmortem neuropathological analysis defines the final diagnosis of MSA by proving α‐synuclein‐positive oligodendroglial inclusions and selective neurodegeneration. Based on the pattern of neurodegeneration, MSA can also be classified into 3 subtypes: MSA with striatonigral degeneration (MSA‐SND), with olivopontocerebellar atrophy (MSA‐OPCA), and a combination of the 2, referred to as mixed.^2^ One of the early signs of MSA is the appearance of respiratory and cardiovascular autonomic disturbances, including orthostatic hypotension (OH).^3^ The drop in blood pressure in patients produces a reduction in blood flow that could lead to a fall of oxygen supply to the brain. Moreover, OH when associated with nocturnal hypertension has been described as a risk factor for cerebral microangiopathy (end‐organ damage), generating events of brain hypoxia/ischemia followed by reperfusion.^4^, ^5^ Furthermore, with the progression of the disease, most of the MSA patients develop sleep and respiratory disorders, like obstructive sleep apnea^6^ and nocturnal stridor,^7^ which lead to a reduction in oxygen saturation by recurrent collapse of the upper airways during inspiration. Autonomic involvement is common in Parkinson's disease (PD) but is more variable in severity than in MS.^8^

Based on all these findings, we therefore hypothesize that, in association with the pronounced respiratory and cardiovascular autonomic disturbances throughout a disease course of 6 to 8 years, MSA patients are likely to suffer events of hypoxia in the brain, which could lead to an aggravation of disease progression as compared with PD.

## 1 Material and Methods

### 1.1 Human Samples

Fresh‐frozen substantia nigra and visual cortex samples were provided by the Queen Square Brain Bank (QSBB) at University College London. The brain donation program and protocols have received ethical approval for donation and research by the NRES Committee London – Central, and tissue is stored for research under a license issued by the Human Tissue Authority (No. 12198). Samples from 18 MSA patients (clinical diagnosis before postmortem evaluation: 8 MSA‐P, 10 MSA‐C; postmortem pathological evaluation: 3 MSA‐SND, 10 MSA‐OPCA, 5 MSA‐mixed), 12 PD patients, and 10 nonneurological controls (C) were included in this study. Demographical and clinical information was kindly provided by the QSBB (Table 1; the case list including all the clinical and pathological details is available in Supplementary Table 1). No personal information could be extracted from these data. The utilization of postmortem human samples was approved by the corresponding biobank ethics committees and by the Ethics Committee of the Medical University of Innsbruck (AN2016‐0012 358/4.2 359/4.1).

**TABLE 1:** Demographic and clinical data of patients with MSA, patients with PD, and C

					Post hoc analysis		
	C (n = 10)	PD (n = 12)	MSA (n = 18)	*P* values, all	MSA vs C	MSA vs PD	PD vs C
Age (years), mean ± SD^a^	83.1 ± 4.63	77.42 ± 5.16	65.11 ± 6.18	< 0.0001	<0.0001	0.002	0.3128
Female, % (n)^b^	60 (6)	41.7 (5)	61.1 (11)	0.5394			
Disease duration (years), mean ± SD^c^	NA	17 ± 7.39	7.39 ± 2.62	< 0.0001			
*HIF2A* mRNA levels in substantia nigra, a.u., mean ± SD^d^	0.021 ± 0.011	0.018 ± 0.005	0.022 ± 0.01	0.3529			
HIF2α protein levels in substantia nigra, a.u, mean ± SD^d^	0.011 ± 0.004	0.014 ± 0.007	0.023 ± 0.008	0.0002	0.0003	0.0084	>0.6688
*HIF2A* mRNA levels in visual cortex, a.u, mean ± SD^d^	0.010 ± 0.008	0.009 ± 0.005	0.014 ± 0.01	0.2197			
HIF2α protein levels in visual cortex, a.u, mean ± SD^d^	0.012 ± 0.012	0.017 ± 0.012	0.021 ± 0.008	0.09	0.0892	0.9413	0.7207

NA, not applicable; SD, standard deviation; a.u., arbitrary units.

A more detailed table including all cases and the clinical and pathological information can be found in Supplementary table 1.

^a^ Kruskal‐Wallis with post hoc Dunn's test.

^b^ Chi‐square test.

^c^ Unpaired *t* test, 2‐tailed.

^d^ One‐way ANOVA with post hoc Bonferroni's test.

### 1.2 Molecular Analyses

Total RNA and proteins were obtained from frozen samples using Trizol Reagent (Thermofisher) according to the manufacturer's instructions. No differences were observed in RNA quality between MSA, PD, and C in the different brain regions, with A260/280 ratios within the expected range (substantia nigra: C, 1.93 ± 0.06; PD, 1.91 ± 0.04; MSA, 1.92 ± 0.05; visual cortex: C, 1.98 ± 0.01; PD, 1.97 ± 0.01; MSA, 1.96 ± 0.03). qRT‐PCR was performed using standard procedures. GAPDH, 18S, ACTB mRNA were used as housekeeping genes. All samples were run in duplicate, and results were analyzed using the 2−ΔCt method^9^ and presented as relative gene expression normalized to the average cycle threshold for the 3 housekeeping genes. Protein quantification and Western blot were performed using standard procedures. Primary antibodies included anti‐HIF2α (Cell Signaling; 7096), anti‐HIF1α (BD; 610959), anti‐GAPDH (Sigma; G9545), anti‐α‐Tubulin (Abcam; ab7750), and anti‐ß‐tubulin (Sigma; T4026) as loading controls. Signal detection was performed using horseradish peroxidase–conjugated antirabbit (Cell Signaling; 7074) or antimouse (GE Healthcare; NA931) antibodies. Images were acquired using the Fusion FX system for Western blot and gel imaging. Relative protein levels were measured by densitometry using FUSION CAPT V16.09b software (Vilber Lourmat) and normalized to the average densiometric value for the 3 housekeeping proteins.^10^ A reference sample was loaded in all gels for gel‐to‐gel normalization. All gels were run, transferred, incubated, and developed in parallel.

### 1.3 Statistical Analyses

Most statistical analyses and all graphs were performed in GraphPad Prism version 8.0 (GraphPad Inc.). Data are expressed as mean ± SD; *P* ≤ 0.05 was considered statistically significant. All individual measurements constituted biological replicates. Samples were evaluated for normal distribution using D'Agostino and Pearson's omnibus normality test. Comparisons were done with 1‐way analysis of variance (ANOVA) with Bonferroni's test for HIF2α protein, *HIF2A* gene expression, and postmortem interval (PMI). A 2‐tailed *t* test was used to analyze disease duration differences between PD and all MSA cases and ANOVA with Bonferroni's test for PD and MSA variants. The Kruskal‐Wallis test with Dunn's test and the chi‐square test were used to evaluate age and sex differences, respectively, between groups. Univariate analysis of variance for HIF2α with covariate age and fix factors diagnosis and sex was performed with SPSS version 24 (IBM) to evaluate the possible confounding effect of age. Correlations were studied using linear regression analysis.

## 2 Results

### 2.1 MSA Brains Show Signs of Chronic Hypoxia

**FIG. 1.**
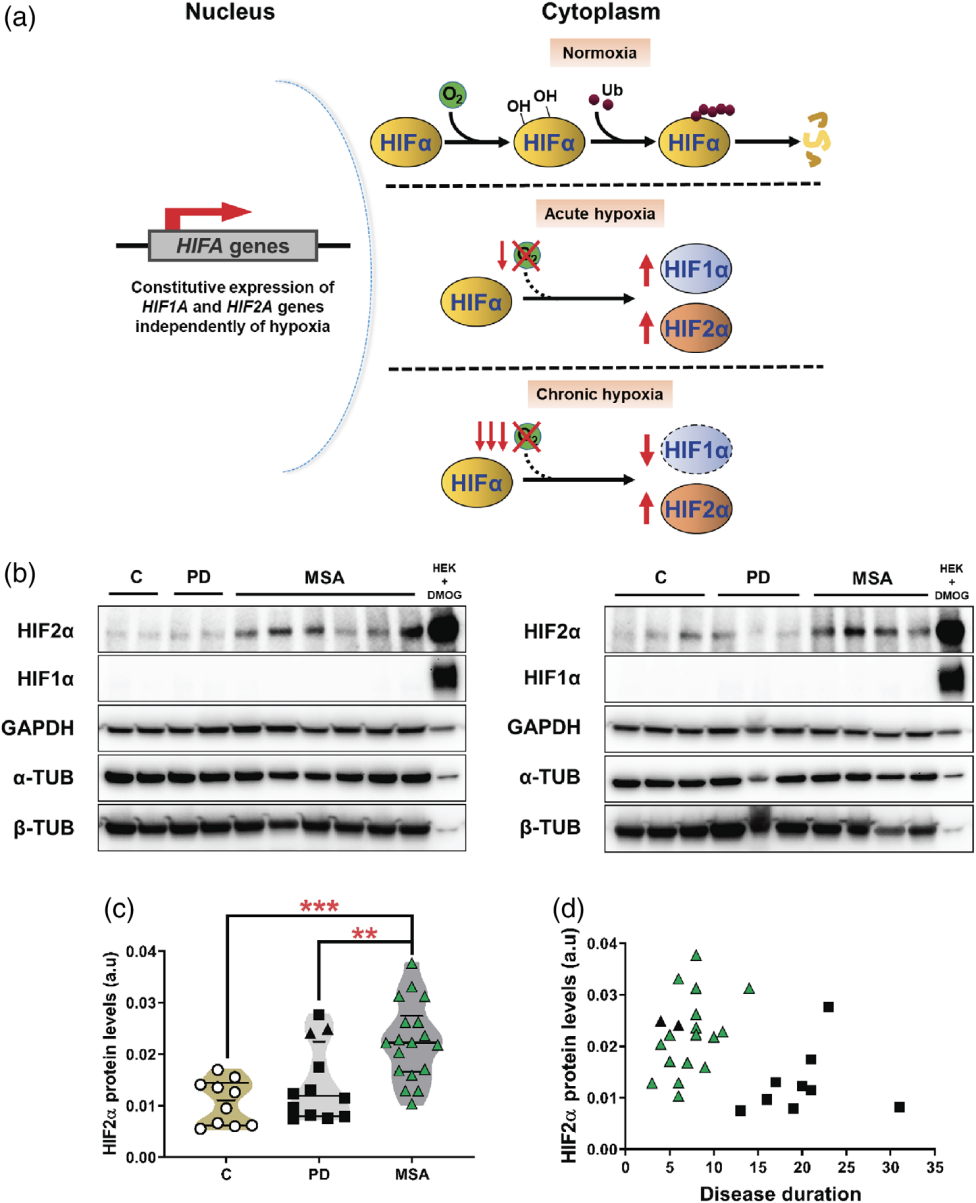
Signs of chronic hypoxia in MSA. (**a**) Schematic representation of the regulation of HIFα in normoxia and acute or chronic hypoxia. Genes coding for HIFα subunits are constitutively transcribed and translated. However, under normal oxygen conditions (normoxia), the resultant proteins are rapidly hydroxylated, ubiquitinated, and degraded via proteasome in an oxygen‐dependent manner. Under acute hypoxic conditions, the decrease in oxygen levels impairs the hydroxylation, which results in HIF1α and HIF2α protein stabilization. Under chronic hypoxic conditions, HIF1α protein is degraded, and HIF2α drives the cellular adaptation. (**b**) Representative Western blot images showing HIF2α and HIF1α protein levels in extracts from human substantia nigra. Samples from control (**c**) and PD and MSA subjects were utilized. GAPDH, α‐tubulin, and β‐tubulin were used as loading controls. A protein extract from HEK cells exposed to DMOG, an agent that mimics hypoxic conditions by inhibiting PHDs, was used as a positive control (right lane). (**c**) Violin plot illustrating HIF2α protein levels in control and PD and MSA cases based on the quantification of Western blots. Controls, white circles; PD, black squares; MSA, green triangles; black triangles, PD cases that were clinically misdiagnosed as MSA. ANOVA, analysis of variance. ***P < 0.001, **P < 0.01 (Bonferroni's test). (**d**) Correlation analysis of HIF2α levels and disease duration in MSA and PD cases. MSA only: *r* ^2^ = 0.1919, *P* = 0.069; PD only: *r* ^2^ = 0.1211, *P* = 0.2677; MSA + PD: *r* ^2^ = 0.1385, *P* = 0.0428. PD, black squares; MSA, green triangles; black triangles, PD cases that were clinically misdiagnosed as MSA. [Color figure can be viewed at wileyonlinelibrary.com]

The hypoxia‐inducible factors (HIFs) are key in the cellular adaptation to low oxygen levels.^11^ These transcription factors are DNA‐binding heterodimers consisting of alpha and beta subunits.^11^ Genes coding for HIFα subunits are constitutively transcribed and translated in normal oxygen conditions, but the resultant proteins are degraded by the proteasome through their consecutive hydroxylation by prolyl‐hydroxylases (PHDs) and ubiquitination by von Hippel‐Lindau ligase in an oxygen‐dependent manner^11^ (Fig. 1A). However, when oxygen availability is scarce, PHDs reduce their activity, and HIFα proteins are stabilized and transported to the nucleus. There HIFα proteins heterodimerize with HIFβ subunits and activate a plethora of different transcriptional programs that are cell specific^12^ (Fig. 1A). HIF1α and HIF2α constitute the 2 main factors driving the cellular adaptation to hypoxia, in which HIF1α is primarily active during the acute phase of hypoxic adaptation, and HIF2α dominates during later, more chronic phases of hypoxia^11^, ^13^, ^14^, ^15^ (Fig. 1A).

Therefore, to evaluate the presence of hypoxia in human brains, we analyzed the protein levels of HIF1α and HIF2α (Fig. 1B). In agreement with the main hypothesis of the present study, Western blot analyses indicated the presence of a hypoxic environment in the substantia nigra of MSA patients compared with PD patients and controls, characterized by the significant accumulation of the hypoxia marker HIF2α (MSA vs C, *P* = 0.0003; MSA vs PD, *P* = 0.0084; Fig. 1B,C and Table 1). The differences between groups remained significant after performing univariate analysis including age as a covariate (MSA vs C, *P* = 0.021; MSA vs PD, *P* = 0.05) and confirmed that changes in HIF2α levels were determined by diagnosis (*P* = 0.022) but not age (*P* = 0.496) or sex (*P* = 0.502), discarding a possible confounding effect. No differences in HIF2α protein levels were observed between MSA variants defined according to clinical symptoms (MSA‐P vs MSA‐C, *P* > 0.9999; Supplementary Fig. 1A and Supplementary Table 2) or postmortem pathological subtype (MSA‐SND vs MSA‐OPCA, *P* > 0.9999; MSA‐SND vs MSA‐mixed, *P* > 0.9999; MSA‐OPCA vs MSA‐mixed, *P* > 0.9999; Supplementary Fig. 1B and Supplementary Table 3). Interestingly, 2 of the PD patients with higher levels of HIF2α were originally misdiagnosed as MSA‐P, suggesting a possible association between the presence of hypoxia and the clinical presentation (Fig. 1C and Supplementary Fig. 1A,B). Furthermore, gene expression analysis showed no differences in *HIF2A* (also known as *EPAS1*) mRNA levels between groups (Table 1, Supplementary Fig. 1C–E, and Supplementary Tables 2–3). This finding indicated that the increase of HIF2α protein was not because of upregulation of the *HIF2A* gene (HIF2α vs *HIF2A*: *r* ^2^ = 0.01613, *P* = 0.4473), but of protein stabilization under hypoxic conditions in MSA. In addition, we evaluated molecular signs of hypoxia in the visual cortex, a brain region less affected in α‐synucleinopathies, in which we observed a numerical increase of HIF2α protein in MSA cases compared with controls, although not significantly (MSA vs C: *P* = 0.0892); see Table 1 and Supplementary Figure 2A,B. As in the substantia nigra, higher HIF2α protein was not associated with upregulation of the *HIF2A* gene (HIF2α vs *HIF2A*: *r* ^2^ = 0.05844, *P* = 0.1329; Supplementary Fig. 2C). We did not detect HIF1α protein in the tissue samples of patients or controls (Fig. 1B and Supplementary Fig. 2A).

That HIF2α levels were significantly higher in MSA cases compared with controls in areas more affected by the pathology may suggest a possible association between hypoxia and disease progression. Therefore, we hypothesized that hypoxia may also have an impact on disease duration in α‐synucleinopathies. However, correlation analysis did not show a significant relationship between disease duration and hypoxia level in MSA (*r* ^2^ = 0.1919, *P* = 0.069; Fig. 1D) or in PD (*r* ^2^ = 0.1211, *P* = 0.2677; Fig. 1D). Nevertheless, the small number of cases per group and the presence of a similar disease duration in most MSA cases may reduce the statistical power of the correlation analysis. A significant negative relationship between hypoxia and disease duration was only observed when cases from both synucleinopathies were pooled together (*r* ^2^ = 0.1385, *P* = 0.0428; Fig. 1D), suggesting that the presence of hypoxia in MSA may at least partly contribute to the faster progression compared with PD. However, the latter may artificially result from the lack of significant change of HIF2α in PD cases, together with a much longer disease duration in PD than MSA.

Finally, correlation analyses of PMI and HIF2α protein levels in the substantia nigra or visual cortex demonstrated that the presence of hypoxia in MSA brains was independent of the time elapsed between death and the freezing of the sampled tissue (substantia nigra: *r* ^2^ = 0.03268; *P* = 0.2643; visual cortex: *r* ^2^ = 0.001221; *P* = 0.8305), therefore discarding PMI as a possible confounding factor.

## 3 Discussion

Here we show that MSA patients present signs of hypoxia in the brain, characterized by the significant accumulation of the hypoxia marker HIF2α compared with PD patients and controls. Based on HIF2α driving the response to chronic hypoxia and being degraded by intermittent hypoxia, whereas HIF1α being primarily active in acute hypoxia and being destabilized under chronic hypoxic conditions^11^, ^13^, ^14^, ^15^, ^16^ (Fig. 1A), our results suggest that MSA patients may suffer from chronic hypoxic events.

The effects of hypoxia in the brains of humans and animal models have been extensively described. Hypoxia has a major effect on oligodendrocytes, inhibiting the proliferation of progenitors, reducing maturation and myelination,^17^, ^18^ and directly inducing oligodendrocyte cell loss and demyelination.^19^ Moreover, in vitro and in vivo studies have shown hypoxia induces brain mitochondrial dysfunction,^20^ oxidative stress,^21^ neuroinflammation,^22^, ^23^, ^24^ and production of reactive oxygen species and proinflammatory cytokines by microglial cells.^25^, ^26^ In summary, in the central nervous system hypoxia induces detrimental effects in processes that are also involved in MSA pathogenesis such as neuroinflammation, neurodegeneration, or demyelination and especially in some particular cell populations affected in this α‐synucleinopathy, that is, oligodendrocytes, microglial cells, or neurons. Therefore, the presence of hypoxia in the brains of MSA patients could lead to an acceleration and aggravation of the pathology acting like a double hit that could explain to some extent the severity and rapid disease course of this fatal neurodegenerative disorder (Supplementary Fig. 3).

Further in vitro and in vivo experiments combining the exposure to hypoxia with the presence of α‐syn will clarify the pathophysiological consequences of hypoxia in α‐synucleinopathy and the cellular and molecular mechanisms underlying these events. We must acknowledge the lack of information regarding the agonal state of all cases included in the study, which could contribute to the generation of hypoxia. However, we would not expect major differences between the groups considering that nonneurological controls suffered from advanced stages of cancer. We also have to acknowledge that whether the hypoxic environment observed in MSA patients is cause or consequence of the disease remains unclear. The use of postmortem samples from end‐stage MSA cases constitutes a limitation because we cannot extract from the current data if hypoxia is present throughout the entire disease course, even at early stages. Nevertheless, based on (1) differences in hypoxia levels between MSA cases and controls being significantly higher in the substantia nigra than in the visual cortex, one of the most affected brain regions in α‐synucleinopathies versus an area less affected; (2) PD cases with a similar clinical presentation to MSA showing signs of hypoxia; and (3) hypoxia levels in the substantia nigra correlating with shorter disease duration (rapid progression) when both α‐synucleinopathies are considered, we concluded that the presence of chronic hypoxia may be associated with the neurodegenerative process underlying α‐synucleinopathies.

Our results suggest a new alternative pathway to understand the pathogenic events underlying α‐synucleinopathies that could provide novel targets for disease modification, especially in MSA. In this regard, the use of supplemental oxygen may constitute a potential therapeutic strategy to mitigate symptoms or even slow the progression of this devastating disease.

## Authors’ contributions

A.H.G.: conception and design, acquisition, analysis and interpretation of data; drafting the manuscript. C.D.: acquisition of data. S.E.: acquisition of data. J.L.H., G.W., and N.S.: conception and design, interpretation of data. All authors read and approved the final article.

## Financial disclosures

Antonio Heras‐Garvin: employed by Medical University of Innsbruck. Christoph Danninger: none. Sabine Eschlböck: employed by Medical University of Innsbruck. Prof. Janice L. Holton: Parkinson's UK Scientific Advisory Board, MSA Trust Scientific Advisory Board; employed by University College London; grants from MSA Trust, CBD Solutions, MRC UK. Prof. Gregor K. Wenning: consultant for and honoraria from Biogen, Biohaven, Lundbeck, Ono, Takeda, UCB; Biogen and Lundbeck advisory boards; royalties from Springer, Cambridge University Press; grants from FWF and MSA Coalition; employed by Medical University of Innsbruck. Prof. Nadia Stefanova: employed by Medical University of Innsbruck; grants form FWF.

## Supporting information


**SUPPLEMENTARY FIG. 1.** HIF2α protein and mRNA levels in MSA variants versus PD and C. (A) HIF2α protein levels in C, PD, and MSA case variants according to the clinical presentation. C, white circles; PD, black squares; MSA, green triangles; black triangles, PD cases that were clinically misdiagnosed as MSA. (B) HIF2α protein levels in C, PD, and MSA case variants according to the postmortem pathological presentation. C, circles; PD, squares; MSA‐SND, red triangles; MSA‐OPCA, blue triangles; MSA‐mixed, purple triangles. (C) *HIF2A* gene expression in C, PD, and MSA. (D) *HIF2A* gene expression in C, PD, and MSA case variants according to the clinical presentation. € *HIF2A* gene expression in C, PD, and MSA case variants according to the postmortem pathological presentation. ANOVA, aalysis of variance. **P < 0.01, *P < 0.05 (Bonferroni's test).

**SUPPLEMENTARY FIG. 2.** HIF2α protein and mRNA levels in the visual cortex of MSA, PD, and C. (A) Representative Western blot images showing HIF2α and HIF1α protein levels in extracts from human visual cortex. Samples from C, PD, and MSA subjects were used. GAPDH, α‐tubulin, and β‐Tubulin were used as loading controls. A protein extract from HEK cells exposed to DMOG, an agent that mimics hypoxic condition by inhibiting PHDs, was used as the positive control (right lane). (B) Violin plot illustrating HIF2α protein levels in C, PD, and MSA cases based on the quantification of Western blots. (C) *HIF2A* gene expression in C, PD, and MSA. C, white circles; PD, black squares; MSA, green triangles; black triangles, PD cases that were clinically misdiagnosed as MSA.

**SUPPLEMENTARY FIG. 3.** Pathophysiological features of MSA and potential pathogenic effect of hypoxia. (A) Schematic overview of the central nervous system in MSA and the different pathogenic processes that could be aggravated by hypoxia. In MSA α‐syn accumulates in the cytoplasm of oligodendrocytes in glial cytoplasmic inclusions (GCIs), inducing oligodendroglial dysfunction. The generation of a hypoxic environment within the central nervous system of MSA patients could increase mitochondrial impairment, leading to the formation of reactive oxygen species (ROS), microglial activation, neuroinflammation, oligodendrocyte dysfunction, and demyelination, aggravating the neurodegenerative process and accelerating disease progression.

**Supplementary table 1:** *Demographic and clinical data of all individuals*.

**Supplementary table 2:** *Demographic and clinical data of patients with MSA‐P, MSA‐C, PD and C*.
**Supplementary table 3**: *Demographic and clinical data of patients with MSA‐SND, MSA‐OPCA, MSA‐mixed, PD and C*.
