# Supplementary figures and images for: Signs of Chronic Hypoxia Suggest a Novel Pathophysiological Event in α‐Synucleinopathies
Source: Mov Disord. 2020 Sep 3;35(12):2333–8. doi: 10.1002/mds.28229 (PMC7818169; doi:10.1002/mds.28229)

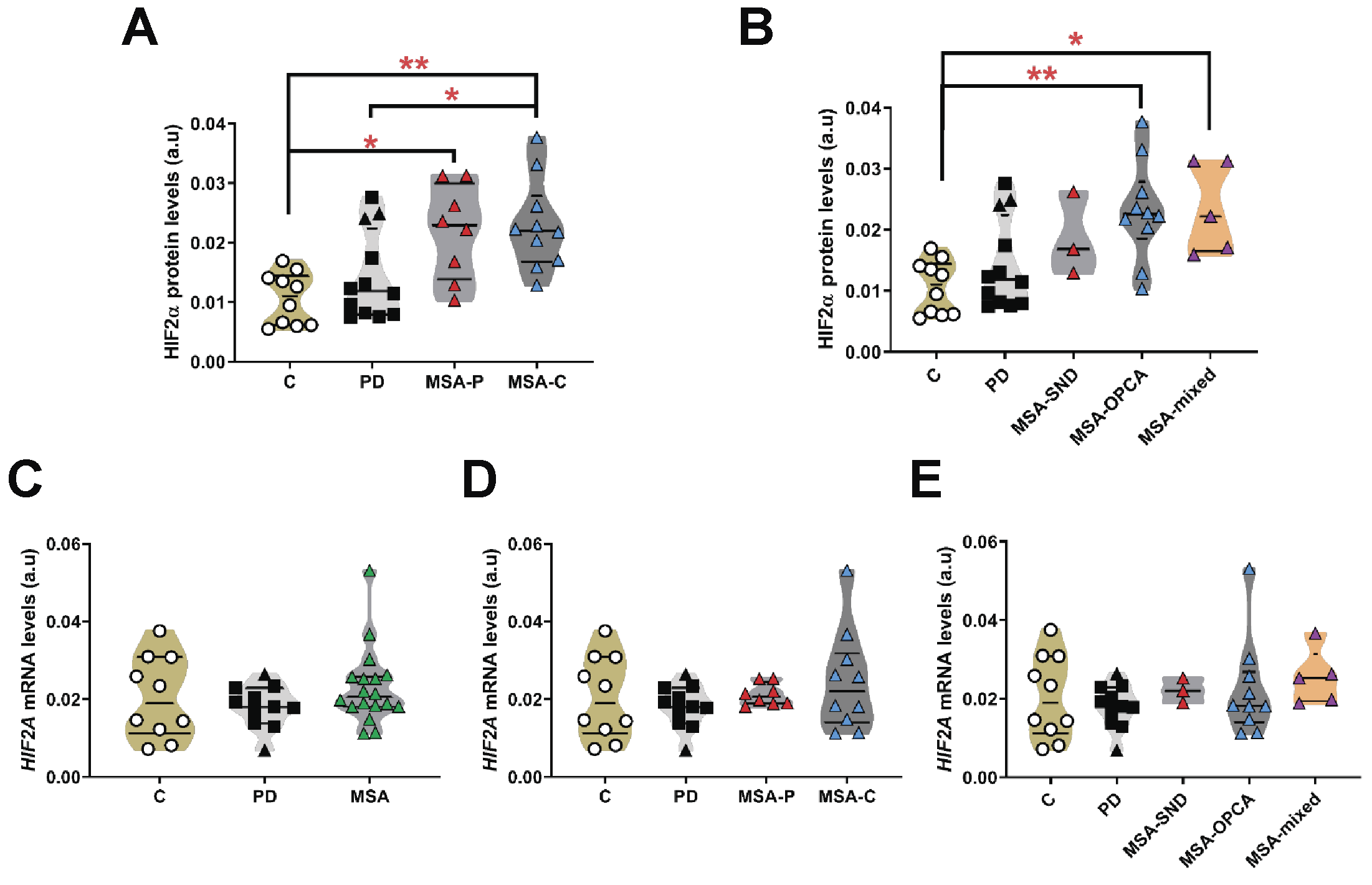

Supplement: Supplementary file 1 — SUPPLEMENTARY FIG. 1. HIF2α protein and mRNA levels in MSA variants versus PD and C. (A) HIF2α protein levels in C, PD, and MSA case variants according to the clinical presentation. C, white circles; PD, black squares; MSA, green triangles; black triangles, PD cases that were clinically misdiagnosed as MSA. (B) HIF2α protein levels in C, PD, and MSA case variants according to the postmortem pathological presentation. C, circles; PD, squares; MSA‐SND, red triangles; MSA‐OPCA, blue triangles; MSA‐mixed, purple triangles. (C) HIF2A gene expression in C, PD, and MSA. (D) HIF2A gene expression in C, PD, and MSA case variants according to the clinical presentation. € HIF2A gene expression in C, PD, and MSA case variants according to the postmortem pathological presentation. ANOVA, aalysis of variance. **P < 0.01, *P < 0.05 (Bonferroni's test). [file MDS-35-2333-s001.tif]

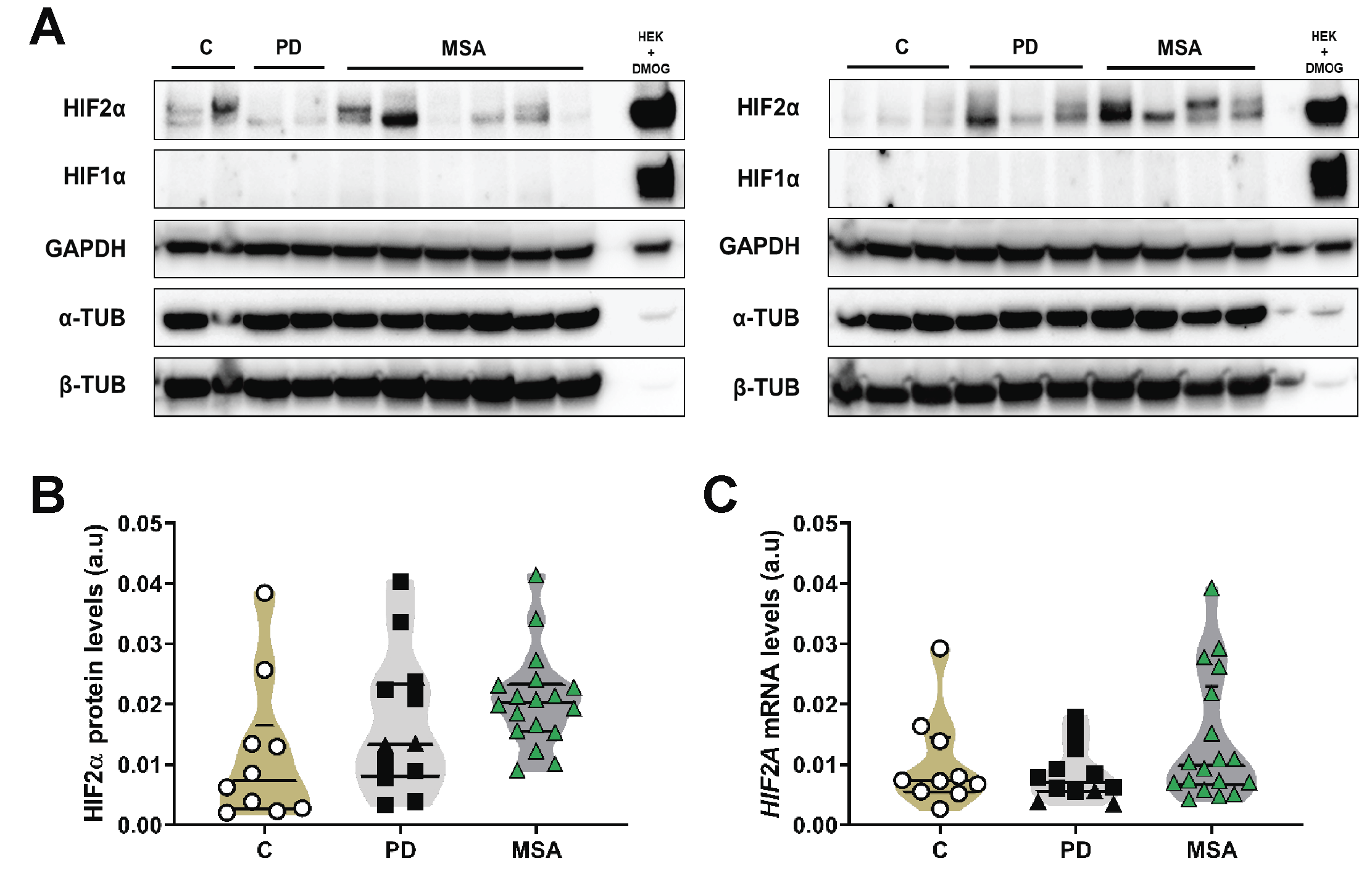

Supplement: Supplementary file 2 — SUPPLEMENTARY FIG. 2. HIF2α protein and mRNA levels in the visual cortex of MSA, PD, and C. (A) Representative Western blot images showing HIF2α and HIF1α protein levels in extracts from human visual cortex. Samples from C, PD, and MSA subjects were used. GAPDH, α‐tubulin, and β‐Tubulin were used as loading controls. A protein extract from HEK cells exposed to DMOG, an agent that mimics hypoxic condition by inhibiting PHDs, was used as the positive control (right lane). (B) Violin plot illustrating HIF2α protein levels in C, PD, and MSA cases based on the quantification of Western blots. (C) HIF2A gene expression in C, PD, and MSA. C, white circles; PD, black squares; MSA, green triangles; black triangles, PD cases that were clinically misdiagnosed as MSA. [file MDS-35-2333-s002.tif]

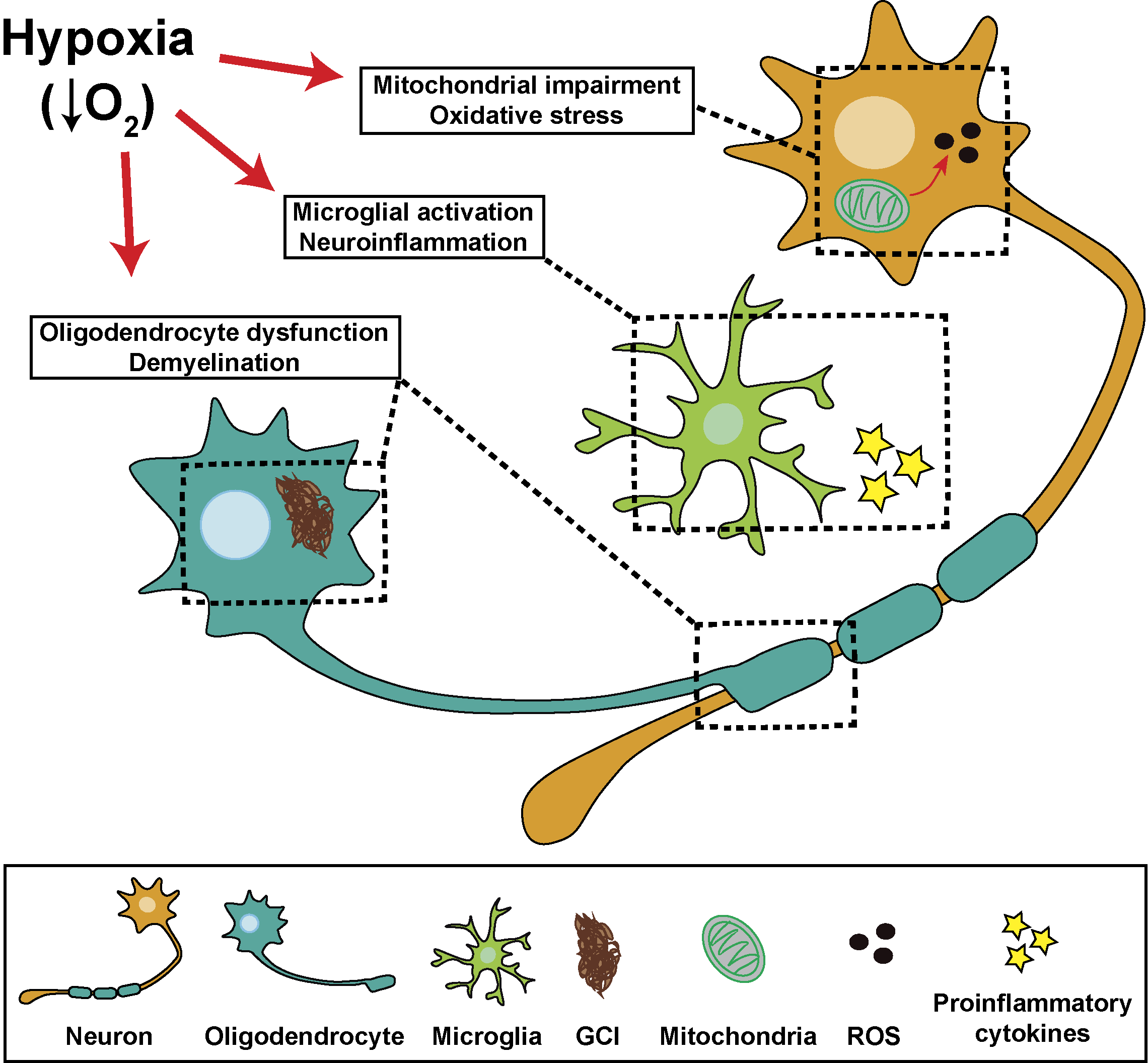

Supplement: Supplementary file 3 — SUPPLEMENTARY FIG. 3. Pathophysiological features of MSA and potential pathogenic effect of hypoxia. (A) Schematic overview of the central nervous system in MSA and the different pathogenic processes that could be aggravated by hypoxia. In MSA α‐syn accumulates in the cytoplasm of oligodendrocytes in glial cytoplasmic inclusions (GCIs), inducing oligodendroglial dysfunction. The generation of a hypoxic environment within the central nervous system of MSA patients could increase mitochondrial impairment, leading to the formation of reactive oxygen species (ROS), microglial activation, neuroinflammation, oligodendrocyte dysfunction, and demyelination, aggravating the neurodegenerative process and accelerating disease progression. [file MDS-35-2333-s003.tif]
