# Supplementary material for: Signs of Chronic Hypoxia Suggest a Novel Pathophysiological Event in α‐Synucleinopathies
Source: Mov Disord. 2020 Sep 3;35(12):2333–8. doi: 10.1002/mds.28229 (PMC7818169; doi:10.1002/mds.28229)
Supplement: Supplementary file 4 — Supplementary table 1: Demographic and clinical data of all individuals. [file MDS-35-2333-s004.docx]

| **Individuals** | **Clinical diagnosis** | **Pathological diagnosis** | **Gender** | **OH** | **OSA** | **Stridor** | **Disease duration** | **Age** | **PMI** |
| --- | --- | --- | --- | --- | --- | --- | --- | --- | --- |
|  |  |  |  |  |  |  |  |  |  |
| **C 1** | Aortic stenosis and left ventricular failure | NNC | F | No record | No record | No record | n.a | 87 | 51.45 |
| **C 2** | Pancreatic cancer | NNC | F | No record | No record | No record | n.a | 80 | 49.10 |
| **C 3** | Squamous cell carcinoma | NNC | M | No record | No record | No record | n.a | 83 | 39.45 |
| **C 4** | Colon cancer | NNC | F | No record | No record | No record | n.a | 86 | 41.05 |
| **C 5** | Cardiac failure | NNC | M | No record | No record | No record | n.a | 87 | 57 |
| **C 6** | Pancreatic cancer | NNC | F | No record | No record | No record | n.a | 79 | 87.50 |
| **C 7** | Metastatic cancer | NNC | F | No record | No record | No record | n.a | 73 | 24.00 |
| **C 8** | Metastatic cancer | NNC | M | No record | No record | No record | n.a | 88 | 97.30 |
| **C 9** | Prostate cancer | NNC | M | No record | No record | No record | n.a | 84 | 55.10 |
| **C 10** | Hypoandrenalism and diabetes | NNC | F | No record | No record | No record | n.a | 84 | 53.35 |
|  |  |  |  |  |  |  |  |  |  |
| **PD 1** | iPD | iPD Braak 6 | F | No record | No record | No record | 21 | 80 | 45.4 |
| **PD 2** | iPD | iPD Braak 6 | F | No record | No record | No record | 23 | 83 | 40.3 |
| **PD 3** | iPD | iPD Braak 6 | F | No record | No record | No record | 31 | 78 | 45.45 |
| **PD 4** | iPD | iPD Braak 6 | M | No record | No record | No record | 16 | 83 | 29.45 |
| **PD 5** | iPD | iPD Braak 6 | F | No record | No record | No record | 17 | 74 | 26.2 |
| **PD 6** | iPD | iPD Braak 6 | M | Yes | No record | No record | 13 | 79 | 27.2 |
| **PD 7** | iPD | iPD Braak 6 | F | Yes | No record | No record | 13 | 76 | 49.1 |
| **PD 8** | iPD | iPD Braak 6 | M | No record | No record | No record | 20 | 64 | 56 |
| **PD 9** | iPD | iPD Braak 6 | M | No record | No record | No record | 19 | 78 | 34.15 |
| **PD 10** | iPD | iPD Braak 6 | M | No record | No record | No record | 21 | 77 | 42.55 |
| **PD 11** | MSA-P | iPD Braak 5 | M | Yes | No record | No record | 4 | 75 | 26.15 |
| **PD 12** | MSA-P | iPD Braak 6 | M | Yes | No record | No record | 6 | 82 | 31.2 |
|  |  |  |  |  |  |  |  |  |  |
| **MSA 1** | MSA-P | SND | M | Yes | Yes | No record | 6 | 62 | 36.4 |
| **MSA 2** | MSA-P | Mixed | F | No record | No record | No record | 14 | 63 | 46.15 |
| **MSA 3** | MSA-P | OPCA | F | Yes | No record | No record | 6 | 66 | 74.2 |
| **MSA 4** | MSA-P | SND | F | Yes | Yes | Yes | 7 | 55 | 33.38 |
| **MSA 5** | MSA-P | Mixed | F | Yes | No record | No record | 5 | 60 | 40.55 |
| **MSA 6** | MSA-P | OPCA | M | None | No record | yes | 8 | 76 | 79.55 |
| **MSA 7** | MSA-P | SND | F | Yes | Yes | No record | 8 | 75 | 36.14 |
| **MSA 8** | MSA-P | Mixed | F | Yes | Yes | No record | 8 | 62 | 24 |
| **MSA 9** | MSA-C | OPCA | F | mild | Yes | Yes | 10 | 65 | 51.15 |
| **MSA 10** | MSA-C | OPCA | M | Yes | No record | No record | 7 | 63 | 41.15 |
| **MSA 11** | MSA-C | Mixed | F | No record | No record | Yes | 9 | 59 | 48.15 |
| **MSA 12** | MSA-C | OPCA | M | yes | No record | No record | 4 | 73 | 85.25 |
| **MSA 13** | MSA-C | OPCA | M | No record | No record | Yes | 8 | 60 | 50.35 |
| **MSA 14** | MSA-C | OPCA | F | Yes | Yes | No record | 6 | 58 | 72.5 |
| **MSA 15** | MSA-C | Mixed | M | Yes | No record | No record | 5 | 72 | 83.55 |
| **MSA 16** | MSA-C | OPCA | F | No record | No record | No record | 3 | 67 | 31.25 |
| **MSA 17** | MSA-C | OPCA | F | No record | No record | No record | 8 | 64 | 83.2 |
| **MSA 18** | MSA-C | OPCA | M | No record | Yes | No record | 11 | 72 | 84.15 |

**Supplementary table 1**: *Demographic and clinical data of all individuals.*

Abbreviations: OH: orthostatic hypotension; OSA: obstructive sleep apnea; PMI: post-mortem interval; C = control; NNC: Non-neurological control; iPD: idiopathic PD; M: male; F: female; n.a: not applicable.
