# Supplementary material for: Signs of Chronic Hypoxia Suggest a Novel Pathophysiological Event in α‐Synucleinopathies
Source: Mov Disord. 2020 Sep 3;35(12):2333–8. doi: 10.1002/mds.28229 (PMC7818169; doi:10.1002/mds.28229)
Supplement: Supplementary file 5 — Supplementary table 2: Demographic and clinical data of patients with MSA‐P, MSA‐C, PD and C. Supplementary table 3: Demographic and clinical data of patients with MSA‐SND, MSA‐OPCA, MSA‐mixed, PD and C. [file MDS-35-2333-s005.docx]

|  |  |  |  |  |  | **Post hoc analysis** | | | | | |
| --- | --- | --- | --- | --- | --- | --- | --- | --- | --- | --- | --- |
|  | **C (n=10)** | **PD (n=12)** | **MSA-P (n=8)** | **MSA-C (n=12)** | **p values, all** | **MSA-P vs HC** | **MSA-P vs PD** | **MSA-C vs C** | **MSA-C vs PD** | **MSA-P vs MSA-C** | **PD vs C** |
| **Age, years, mean ± SD ^A^** | 83.1 ± 4.63 | 77.42 ± 5.16 | 64.88 ± 7.26 | 65.3 ± 5.58 | <0.0001 | 0.0002 | 0.0340 | <0.0001 | 0.0182 | >0.9999 | 0.6256 |
| **Female, % (n) ^B^** | 60 (6) | 41.7 (5) | 75 (6) | 50 (5) | 0.5017 |  |  |  |  |  |  |
| **Disease duration, years, mean ± SD ^C^** | NA | 17 ± 7.39 | 7.75 ± 2.77 | 7.1 ± 2.6 | 0.0001 | NA | 0.0016 | NA | 0.0004 | >0.9999 | NA |
| **HIF2A mRNA levels in substantia nigra, a.u, mean ± SD ^C^** | 0.021 ± 0.011 | 0.018 ± 0.005 | 0.021 ± 0.003 | 0.025 ± 0.013 | 0.4460 |  |  |  |  |  |  |
| **HIF2α protein levels in substantia nigra, a.u, mean ± SD ^C^** | 0.011 ± 0.004 | 0.014 ± 0.007 | 0.022 ± 0.008 | 0.023 ± 0.008 | 0.0007 | 0.0102 | 0.1377 | 0.0019 | 0.0351 | >0.9999 | >0.9999 |

**Supplementary table 2**: *Demographic and clinical data of patients with MSA-P, MSA-C, PD and C.*

Abbreviations: NA = not applicable; SD = standard deviation; a.u = arbitrary units.

A. Kruskal-Wallis with post hoc Dunn’s test

B. Chi-square test.

C. One-way ANOVA with post hoc Bonferroni’s test.

|  |  |  |  |  |  |  | **Post hoc analysis** | | | | | | | | | |
| --- | --- | --- | --- | --- | --- | --- | --- | --- | --- | --- | --- | --- | --- | --- | --- | --- |
|  | **C (n=10)** | **PD (n=12)** | **SND (n=3)** | **OPCA (n=10)** | **mixed (n=5)** | **p values, all** | **SND vs C** | **SND vs PD** | **OPCA vs C** | **OPCA vs PD** | **mixed vs C** | **mixed vs PD** | **SND vs OPCA** | **SND vs mixed** | **OPCA vs mixed** | **PD vs C** |
| **Age, years, mean ± SD ^A^** | 83.1 ± 4.63 | 77.42 ± 5.16 | 64 ± 10.15 | 66.4 ± 5.76 | 63.2 ± 5.17 | <0.0001 | 0.0187 | 0.3623 | 0.0005 | 0.0869 | 0.0006 | 0.0482 | >0.9999 | >0.9999 | >0.9999 | >0.9999 |
| **Female, % (n) ^B^** | 60 (6) | 41.7 (5) | 66.6 (2) | 50 (5) | 80 (4) | 0.4982 |  |  |  |  |  |  |  |  |  |  |
| **Disease duration, years, mean ± SD ^C^** | NA | 17 ± 7.39 | 7 ± 1 | 7.1 ± 2.5 | 8.2 ± 3.7 | 0.0005 | NA | 0.0388 | NA | 0.0009 | NA | 0.0239 | >0.9999 | >0.9999 | >0.9999 | NA |
| **HIF2A mRNA levels in substantia nigra, a.u, mean ± SD ^C^** | 0.021 ± 0.011 | 0.018 ± 0.005 | 0.022 ± 0.003 | 0.022 ± 0.012 | 0.0225 ± 0.007 | 0.6535 |  |  |  |  |  |  |  |  |  |  |
| **HIF2α protein levels in substantia nigra, a.u, mean ± SD ^C^** | 0.011 ± 0.004 | 0.014 ± 0.007 | 0.019 ± 0.007 | 0.023 ± 0.008 | 0.024 ± 0.007 | 0.0013 | 0.8933 | >0.9999 | 0.0030 | 0.0546 | 0.0176 | 0.1724 | >0.9999 | >0.9999 | >0.9999 | >0.9999 |

**Supplementary table 3**: *Demographic and clinical data of patients with MSA-SND, MSA-OPCA, MSA-mixed, PD and C.*

Abbreviations: NA = not applicable; SD = standard deviation; a.u = arbitrary units.

A. Kruskal-Wallis with post hoc Dunn’s test

B. Chi-square test.

C. One-way ANOVA with post hoc Bonferroni’s test.
